# How social network analysis can be used to monitor online collaborative learning and guide an informed intervention

**DOI:** 10.1371/journal.pone.0194777

**Published:** 2018-03-22

**Authors:** Mohammed Saqr, Uno Fors, Matti Tedre, Jalal Nouri

**Affiliations:** 1 Department of Computer and System Sciences (DSV), Stockholm University, Kista, Stockholm, Sweden; 2 Qassim University, College of Medicine, Qassim, Melida, Kingdom of Saudi Arabia; 3 School of Computing, University of Eastern Finland, Joensuu, Finland; Tokyo Institute of Technology, JAPAN

## Abstract

To ensure online collaborative learning meets the intended pedagogical goals (is actually collaborative and stimulates learning), mechanisms are needed for monitoring the efficiency of online collaboration. Various studies have indicated that social network analysis can be particularly effective in studying students’ interactions in online collaboration. However, research in education has only focused on the theoretical potential of using SNA, not on the actual benefits they achieved. This study investigated how social network analysis can be used to monitor online collaborative learning, find aspects in need of improvement, guide an informed intervention, and assess the efficacy of intervention using an experimental, observational repeated-measurement design in three courses over a full-term duration. Using a combination of SNA-based visual and quantitative analysis, we monitored three SNA constructs for each participant: the level of interactivity, the role, and position in information exchange, and the role played by each participant in the collaboration. On the group level, we monitored interactivity and group cohesion indicators. Our monitoring uncovered a non-collaborative teacher-centered pattern of interactions in the three studied courses as well as very few interactions among students, limited information exchange or negotiation, and very limited student networks dominated by the teacher. An intervention based on SNA-generated insights was designed. The intervention was structured into five actions: increasing awareness, promoting collaboration, improving the content, preparing teachers, and finally practicing with feedback. Evaluation of the intervention revealed that it has significantly enhanced student-student interactions and teacher-student interactions, as well as produced a collaborative pattern of interactions among most students and teachers. Since efficient and communicative activities are essential prerequisites for successful content discussion and for realizing the goals of collaboration, we suggest that our SNA-based approach will positively affect teaching and learning in many educational domains. Our study offers a proof-of-concept of what SNA can add to the current tools for monitoring and supporting teaching and learning in higher education.

## Introduction

With the advent of the Internet, the use of information technology in education has become increasingly commonplace, and computer-supported collaborative learning (CSCL) has gained grounds in online learning environments [1, 2]. One of the most common implementations of CSCL is the asynchronous discussion board (forum). Forums offer learners the opportunity to collaborate, cooperate, and interact online in themed discussions, as well as the convenience of transcending the physical barriers of time and place [1–4]. The written nature of the contributions in online forums enables explicit writing, reflection, and permanent access to submissions [2].

The benefits of online collaborative learning are supported by a growing body of evidence environments [1, 2]. Online collaborative learning has been associated with higher academic achievement, deeper levels of learning, retention of learned information for longer times, better problem solving, and higher-order critical thinking skills [5–8]. However, working online together does not necessarily mean collaboration [4, 6, 9–12], and offering the learners the opportunity to interact in CSCL does not directly translate to effective collaboration [5, 6, 11]. Common barriers to effective collaboration include social loafing, dysfunctional group dynamics, lack of appreciation of values, absence of a stimulating task or script, lack of preparation, and lack of social skills [7, 10, 11, 13, 14]. For successful online collaboration to take place, there should be active coordination of group dynamics [11], mutual engagement of the learners, discussion moderators [6, 10, 15], scaffolding by instructors [11, 13, 15], and a stimulating environment that maximizes efficient interactions among participants [5, 12].

To ensure that forums meet their intended pedagogical goals (are actually collaborative and stimulate learning), mechanisms are needed for monitoring the efficiency of online collaboration; such methods commonly fall under collaboration analysis [4, 10, 16–19]. Computer based interaction analysis (IA) is one such method that uses data drawn from participants’ activities in order to understand computer-mediated activities and interactions. After its inception, IA was often used to help regulate students’ actions, such as the mode and degree of participation, or to increase awareness of student activity. For teachers, it is used as a tool for supporting decision-making regarding moderation tactics, for anticipating potential problems, and for assessing students’ participation [19, 20]. IA falls short of relational and social aspects. For instance, IA statistics do not offer information about the patterns of interactions, structure of the group, active or inactive participants, influential students, teacher’s role, the patters of flow of information, group dynamics or cohesion, or timeline of interactions [4, 16–18, 21, 22]. Lately, a growing body of research has demonstrated the practicality of using Social Network Analysis (SNA) in offering valuable insights about the social structure, collaborative patterns and roles and position of collaborators [22–24].

### Social network analysis

Social network analysis is a method for studying the structure of relationships and the effect this social structure has on the attitudes, behavior, and performance of the individual actors or groups [25]. A social network has two fundamental elements: *nodes* (network actors or participants) and *edges* (ties or relations) connecting them [25]. In CSCL, nodes represent students and teachers or other actors, and edges represent the interactions or other ties among them. Networks are visually represented by mapping edges (interactions) among nodes (actors) in a special graph commonly known as a “sociogram”. Each node in the network is represented by a circle, and each interaction is represented by an arrow or a line from the source to the target node [25].

The use of SNA visual analytics, along with quantitative network analysis (centrality measures), may broaden the understanding of the properties of online collaboration and the collaborators [23, 24]. SNA complements the level of activity indicators commonly obtained by computer-based IA with insights into three main areas; 1) position in information exchange/collaborative knowledge construction [8, 22, 26–28]; 2) role identification and relational insights [16, 23, 29–31]; and 3) group properties, cohesion and dynamic evolution of relations [32, 33].

A typical use of SNA in collaboration is the of analysis student’s position and role in collaborative knowledge sharing. A preferential student position might positively affect his or her learning and academic achievement—a concept that has garnered a considerable volume of SNA research and explanations [28, 34]. According to the social capital theory, well-connected students have better access to resources and to emotional and educational support, which can boost their sense of belongingness and motivation [35, 36]. Students who connect or mediate communications have another form of social capital; brokerage social capital. Besides having control over the flow of information, they also have privileged access to varying points of view. According to Kranton, Pfeffer [28], individuals who connect to otherwise unconnected groups of collaborators (structural holes) may provide novel ideas and have a better chance of success.

Wenguang, Xinhui [34] demonstrated that students with brokerage positions were important to knowledge construction networks in an online course. Similar results were reported by Gunawardena, Flor [37], who described how students’ positions facilitated information processing and the dissemination of critical knowledge among peers, which benefited the whole groups and assisted relatively peripheral students to make substantive contributions. Zhang and Zhang [27] found that the level of knowledge construction in the studied course was minimal because few students acted as conduits for information exchange in the learning network. They recommended taking measures to improve the quality of interaction and students’ positions in the learning networks. Similar recommendations were reported by Heo, Lim [8] after studying knowledge construction in project-based learning.

Another perspective that can be revealed by using SNA in collaboration analysis is the identification of roles through visual or mathematical analysis [centrality measures) [16, 29, 31]. Rabbany, Takaffoli [16] have demonstrated how SNA can be used to study the structure of online communities, identify active and inactive students, and detect students who are central to the flow of information in discussion forums. Their method can be used to guide the instructor and students about the flow of the course. Similarly, Bakharia and Dawson [30], and Lockyer, Heathcote [38] used SNA to identify interactive and isolated students. Additionally, their research extended to identifying the emergence and evolution of undesirable instructor roles during knowledge sharing where interactions are dominated by instructors albeit being expected to be distributed among participants. Recently, Marcos-García, Martínez-Monés [29] used *SAMSA*, a tool that combines SNA visualization along with interaction analysis to demonstrate the possibility of automatic identification of different collaborative roles and thereafter offer a method for supporting those roles tailored to participants’ needs.

Roles minimize the inert knowledge problem and help collaborators approach the task from different perspectives as well as appreciate different points of view [39]. Preset roles may minimize conflicts and counterproductive self-assignment of undesired roles [40]. On the group level, roles help facilitate intragroup coordination, enhance group cohesion and support participants’ responsibility. Therefore, roles can be used to promote accountability and positive interdependence among collaborators [33]. Using roles within the framework of collaboration scripts is a popular method to prompt engagement in cognitive and socially meaningful interactions [14, 20, 39, 40]. Roles are usually predefined by assigning certain duties or responsibilities to collaborators; Nonetheless, they can also emerge during group interaction [29, 36].

The third construct that SNA may reveal about collaborative groups is cohesiveness, which is measured by density of interactions and clustering coefficient. Density is a measure of group cohesion and diversity of contributions. In contrast to simple quantification of interactions, density is a relative measure that increases when more members participate. Dense groups are more socially cohesive, share different points of view, and show higher levels of satisfaction and stability [22, 33, 34]. Cohesion and interdependence are key concepts of collaborative learning and monitoring group density can uncover an important aspect of collaborative behavior on the group level [33].

The use of monitoring strategies can inform educators about the status of collaboration and allow them to take data driven interventions when needed [14, 20, 22]. SNA visual analytics, along with quantitative SNA indicators have the potential to offer insights on both quantity and quality of collaboration as well as the role of collaborators. The possibility of monitoring online interactions in real-time might open new frontiers for the study of collaboration and how events evolve online [17, 23]. Although research in the field of SNA in education dates back to the late 1990s, studies in education have only focused on the potential of using SNA, but not on the actual benefits they achieved [8, 16, 22, 23, 26, 27, 30, 31, 38, 41–44]. The recent SNA reviews by Cela, Sicilia [23] who reviewed 37 studies and Dado et al. who reviewed 89 studies [24] reported no studies about actual interventions in a learning setting. The study we report on here investigated how social network analysis can be used to monitor online collaborative learning, find aspects in need for improvement, guide an informed intervention, and assess the efficacy of intervention using an experimental, observational repeated measurement design in three courses over a full-term duration.

This study was guided by the overall research question: How can social network analysis be used to guide a data-driven intervention in online collaborative learning as well as assess the efficacy of the intervention?

## Methods

### The context

Qassim University, College of Medicine in Saudi Arabia uses CSCL as a means to enhance clinical reasoning and critical thinking skills in an online collaborative environment [13, 45, 46]. In the three studied courses, teachers use online clinical case scenarios, the cases are posted and moderated by the teacher through CSCL, and students are encouraged to engage in collaborative discussions regarding the case. The case scenarios describes certain medical conditions in terms of hypothetical patient cases with a description of the history, symptoms, clinical findings, and sometimes laboratory investigations or radiological findings. The idea is to stimulate the students through the case to discuss the patient symptoms, reasoning of clinical findings, diagnosis and management. For example, a case in the emergency course described a patient who presented to the emergency department with a cloudy consciousness following a severe headache episode. Students were asked to discuss the case diagnosis, investigations, and treatment. In the surgery course, a case described a child with a cleft palate, students were asked to discuss the possible risk factors, symptoms and management. Interaction analysis indicators reported by the learning management system (Moodle) have shown that a number of clinical courses were highly active in terms of the number of posts, topics, and participating students. However, a pilot study performed during the academic year 2013–2014 concluded that the pattern of online interactions in the studied courses was not as collaborative as hoped. The interactions were mostly instructor-centric, student-student interactions were scarce, most posts were repetitions of answers, and there were few discussions or reflections among participants [31]. Based on these findings, we designed a research plan divided into three stages:

Stage 1: Establish a monitoring mechanism that monitors CSCL and identifies areas of need for intervention in three courses in the first mid-term.
Stage 2: Build an intervention plan based on the analysis of data derived from Stage 1.
Stage 3: Compare post-intervention to pre-intervention and evaluate the whole experiment.

### Subjects

Three courses were involved: Surgery A (course A), Surgery B (course B), and Emergency (course C). The three courses were almost a full term in duration. The teaching method in these courses was blended learning, where the online component included clinical case discussions moderated by the course organizer. Table 1 details the number of students, number of posts, and topics in each course.

**Table 1:** Characteristics of courses and subjects involved in the study.

Course	Participants	Posts	Topics
Course A	17	634	20
Course B	33	1396	21
Course C	32	390	3
Total	82	2420	44

### Study design

The study design followed an experimental, observational repeated measurement design approach. In the first midterm, subjects were monitored using real-time social network analysis; the data were collected by the end of first mid-term and subsequently analyzed. An intervention plan was then formulated based on the analysis of the first mid-term data, and at the end of the second term, data from both terms were compared and analyzed.

### Data collection

Interaction data were extracted using two methods: *Graphfes* web service and *Structured Query Language (SQL)* queries. *Graphfes* was used for the monitoring of CSCL in the learning management system. Graphfes is a web service that extracts interaction data from Moodle forums and generates SNA visualizations of course interactions [17]. It has the advantage of being feasible to implement, fast, and capable of instant rendering of interactions [17, 18]. Although *Graphfes* was useful for getting an overall instant view of all course interactions, the final results required more in-depth analysis and extraction of metadata about the users, their attributes, and the properties of the messages, which necessitated the use of custom SQL queries.

SQL was used to extract detailed interaction data (subject, content, ID, parent forum, author, reply, author of the reply, creation time, modification time, group ID) and user profile data (user ID, name, course, email). The data were then cleaned by removing incomplete, missing, or garbled records (6 records were missing the source or target of interaction). Personal data that could identify users were deleted, and students and participants were coded. The students were coded as G1 to G17 in course A, S1 to S33 in course B, and L1to L32 in course C. The teachers were coded Q1, T1 and D1. Finally, the data were converted to a format compatible with Gephi.

*Gephi* 0.9.1 was chosen for both visualization and network quantitative analysis (centrality measures). Gephi is an open source application with clustering, filtering, and partitioning capabilities [47]. It has several built-in visualization engines and can create animated dynamic network visualizations, which enabled us to study the evolution of interaction patterns before and after the intervention. The visualization was done using Gephi’s default layout algorithm (Forced Atlas 2), which renders each node’s position according to its relations and connections, and has the capability to update the structure according to the time of the interaction [48].

### Data analysis

For the purpose of analysis in this study, we considered two types of indicators, IA and SNA, on both the individual and the group level. Regarding interaction analysis on the group level, we included number of posts, number of topics, and average contribution of each member. On the individual level, the number of contributions, replies, and received interactions were included. These parameters give an overview about the status of interactivity in the group and the contribution of members.

Regarding SNA, on the individual level, the centrality measures relevant to collaborative knowledge sharing were included, these SNA parameters represent the constructs discussed in detail earlier: the level of activity, position in information exchange, and role in the group. On the group level we used group SNA indicators of interactivity and group cohesion.

**Level of activity (Quantity of participation parameters)**: The level of activity of a collaborator was measured by three centrality measures: The out-degree, the in-degree and the degree centrality. In principle, the SNA level of activity indicators may be considered IA indicators as they are measuring the same concept. *Out-degree centrality* was used to indicate the quantity of participants’ interactions, and was calculated by counting all interactions by a participant[49, 50]. A more important measure regarding the size used was *in-degree centrality or influence*, which is the number of interactions a participant receives. A participant usually receives an interaction when he contributes knowledge that is beyond what has been contributed by others, or a point of view that merits discussion, or receives an argument or a reply. In-degree centrality is an indication of influence and quality of contributions as voted by peers [51, 52]. In knowledge exchange contexts, higher in-degree centrality can be considered a sign of expertise, popularity, or leadership. The third measure of size was *degree centrality*, which is the total number of contributed (*out-degree)* and received (*in-degree)* interactions [51, 53]. Degree centrality is another interactivity indicator that takes into account the both directions of interactions.

**Position in information exchange**: The role in information transfer was measured by three measures. The first was *betweenness centrality*; the number of times a participant played a role in coordinating interactions among otherwise unconnected collaborators [49, 50]. Betweenness centrality is an indication of involvement in relaying arguments or argumentations in a forum. It is considered by some as an indicator of influence on the information exchange [28, 54]. Higher betweenness centrality translates to a higher brokerage capital, and possessing a brokerage capital is a sign of creativity and prominence in spreading information and ideas in a network [22, 28, 35]. Lower values of betweenness centrality are a sign of difficulty in or indifference to reaching out to other members of the group without an intermediary or a mediator. The second indicator was *closeness centrality*, which is an indication of how close the user is to other participants—in other words, easy to reach and interact with. Higher values of closeness centrality reflect a reachable position in information exchange, and lower values can be viewed as a sign of social isolation and poor communications [27, 34, 49, 53]. The third indicator was *information centrality*, which is an indicator of the amount of information flowing through a participant in a social network; having a position through which information flows is a privileged asset during information exchange [30, 50, 55].

**Role in the group**: For role identification, our analysis used a combination of visual analysis and centrality scores to label different roles of the participants [16, 29, 50]. The method is based on Marcos-García et al.’s detailed description of each role [29]. They specified three roles for the teacher: guide, facilitator, and observer. The case scenarios used in the three courses in our study required a facilitator role; a facilitator teacher monitors discussions, answers queries when required, supports collaborators with access to resources, and moderates conflicts. According to Marcos-García, Martínez-Monés [29], the SNA criteria for identifying a facilitator teacher based on these aforementioned duties are moderate participation levels (out-degree), reachability (closeness centrality), moderate or low influence (in-degree), medium to high mediation (betweenness centrality). Combining the SNA criteria with visualization helps clarify the relations and dynamics of the role. Regarding student role, Marcos-García, Martínez-Monés [29] identify a range of student roles ranked according to activity level (leader, coordinator, animator, active, peripheral, quiet and missing). Since our case scenarios are flexible, we expect students to be active (participatory), and we expect the emergence of other roles by some students such as coordinators (mediate discussions and partake in argumentations) and leaders (highly active, encourage others and mediate discussions). The SNA criteria for identifying these roles are as follows:

- **Leader**: A high level of activity (moderate to high out-degree, in-degree and degree centralities), an active role and good position in information transfer (moderate to high levels of betweenness centrality and closeness centrality.
- **Coordinator**: A moderate level of activity (moderate out-degree, in-degree and degree centralities), beside a good coordination position (moderate to high levels of betweenness centrality and closeness centrality).
- **Active**: The active role can be *participatory (AP)*, with moderate level of activity and interaction with other students, beside a moderate to low position in information exchange. An active non-participatory (ANP) role has minimal interactivity with other members (moderate to high levels of out-degree and very low in-degree levels).
- **Peripheral**: Low activity, low in-degree as well as a limited role in information exchange.

As for SNA parameters on the group level, we included group interactivity parameters (average in-degree, average out-degree and average degree). The average degree was calculated by dividing the total degree of all participants by the group size (the arithmetic mean), the average in-degree and average out-degree were calculated in the same way. Group cohesion was measured by density of interactions and clustering coefficient. Density is a measure of group cohesion and collaborative behavior that was discussed in details earlier while the average clustering coefficient is a measure of each group member tendency to interact with others. Since group SNA interactivity parameters overlap with IA indicators, for simplicity, we will report the SNA indicator in case of a duplicate construct.

## Research ethics

This research was approved by the Medical Research Ethics Committee of Qassim University, College of Medicine. An online privacy policy that details possible use of data for research and user protection guarantees was signed by all participants. Learners’ data were anonymized, identifying and personal information was masked, and all personal or private information were excluded from analysis. The College privacy guidelines and policies of dealing with students’ data were strictly followed. It is worth noting that using the LMS is neither graded nor mandatory and the authors of this study did not participate in teaching or examination in any of the courses involved in the study.

## Results

### The first mid-term (pre-intervention stage)

#### Visual analysis

**Fig 1:**
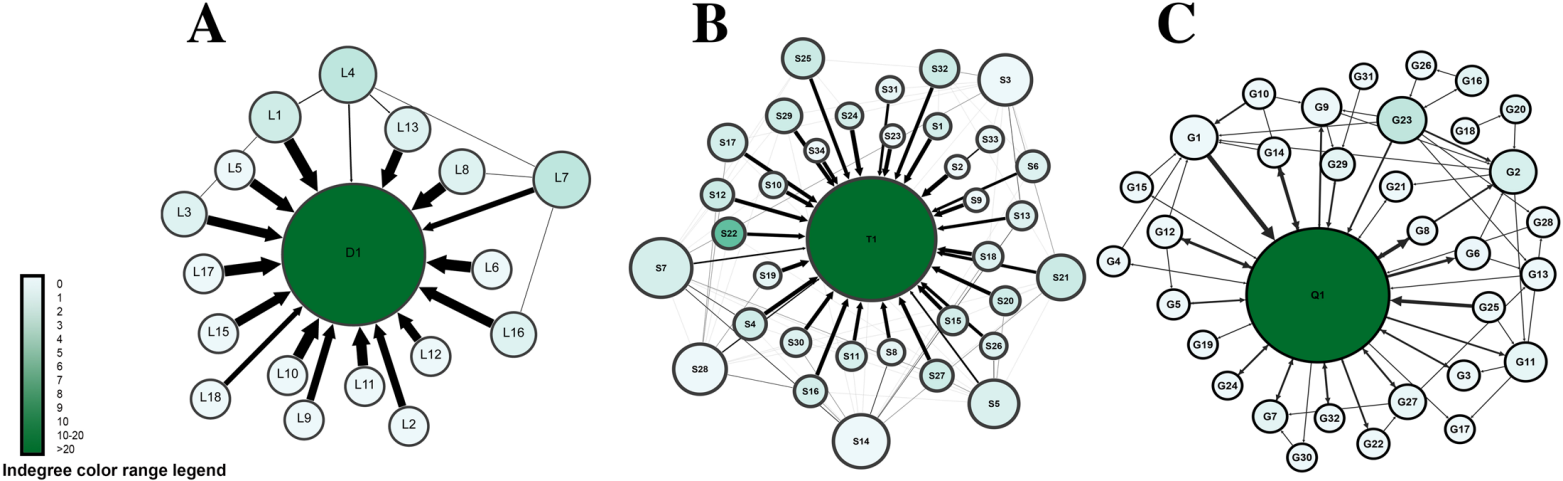
Summary of all interactions in the three courses. Each circle (node) corresponds to a participant. Node size is proportional to the degree centrality (sum of incoming and outgoing interactions). Arrows between nodes represent interactions, and the thickness of the arrows represents frequency of interactions. The arrow heads point to the target of interaction. The color range corresponds to betweenness centrality.

By the end of the first mid-term, data were collected and analyzed by means of social network analysis. Fig 1 presents a sociogram of all interactions in the three courses. The sociograms were configured so that size of the node corresponds to the total number of interactions (degree centrality). The sociograms showed the following:

1. Most interactions were targeting the teacher (an instructor-centric pattern), seen as arrows pointing towards teacher nodes, and as thick arrows (frequent interactions). This was most obvious in course A, followed by course B, and then C. In a collaborative environment, we expect a facilitator teacher or a moderator in a non-dominating role.
2. Student-to-student interactions were scarce, as demonstrated by very few inter-connections between student nodes in course A, very few interconnections in course B, and few thin lines in course C. The paucity of student interactions is another sign of poor collaboration.
3. The degree centrality (sum of incoming and outgoing interactions) of teachers was far larger than for any student, seen as larger teacher nodes compared to those of the students. Teacher-to-student interactions were scarce, except for course C, which showed moderate interactions, seen as bidirectional arrows; an example can be seen in Q1-G8, Q1-G12, Q1-G14, Q1-G24 and Q1-G32. A combination of high degree centrality and few students’ connections means teacher received many interactions, yet they replied infrequently. That implies that students might not have received enough feedback from teachers.
4. Teachers had high betweenness centrality (dark green colored nodes), compared to low betweenness centrality of students (light nodes), which indicates that students played little role in relaying information or connecting others in conversations.

**Fig 2:**
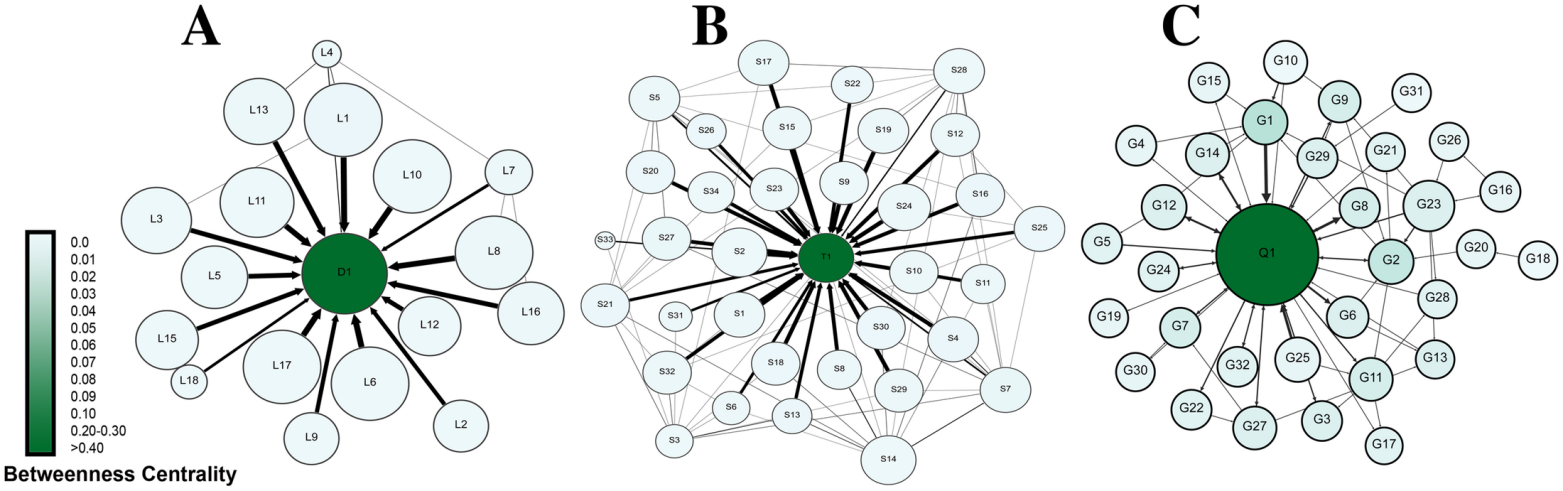
A sociogram of information giving network. Each circle (node) corresponds to a participant. Node size is proportional to the out-degree centrality (sum of outgoing interactions). Arrows between nodes represent interactions, and the thickness of the arrows represents frequency of interactions. The arrow heads represent the target of interaction. The color range corresponds to in-degree centrality.

To demonstrate the *information giving network*, the size of nodes was configured by out-degree centrality (outgoing interactions), where students with more posts have larger nodes. The sociogram in Fig 2 shows that nodes corresponding to students were larger than those in Fig 1. In course A and B many student nodes were larger than the instructor node, indicating that the students wrote more posts than the instructor did. However, the color of the nodes, which was configured to reflect in-degree centrality, shows many very light colored nodes, which indicates that students only gave information or answered queries and had not received interactions from neither peers nor the instructors, so they neither negotiated their arguments, debated others nor got feedback from the instructor.

**Fig 3:**
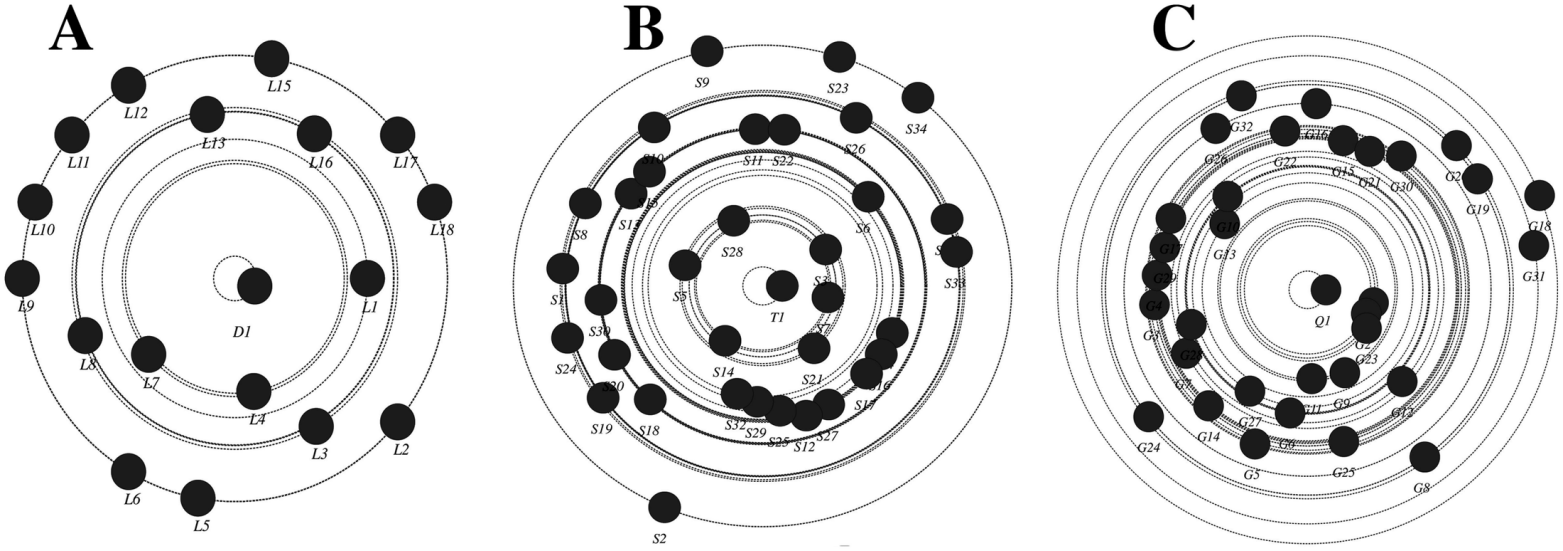
Information centrality plot of the three courses. The closer the node is to the center, the more important role it has in information transfer, so D1, T1 and Q1 (the teachers) are the center of information transfer in the networks.

To better visualize information transfer across the network, we plotted information centrality graph in Fig 3. Information centrality is a measure of each participant’s role in information transfer (how much information traffic passes through a node). The nodes close to the center have a leading role in information transfer, and more distant nodes have more minor roles. The three plots show a common pattern, where the instructors D1, T1, and Q1 occupy a central spot and students lie few steps from the center, indicating that the instructors were dominating rather than facilitating the discussions.

#### Quantitative analysis

Looking at the interaction analysis parameters (number of posts and topics in Table 1, and the average degree and out-degree centralities in Table 2) shows that the three courses were active on both group and learner level. However, looking at the SNA centrality measures in Table 2 and the roles students played in discussions in Table 3, a different picture unfolds. While the level of activity was high as measured by out-degree or number of posts, in Course A each student received a reply for each 35 posts, but in Course B the ratio was one to seven. This significant imbalance is a sign of absence of interactions among students and poor collaboration. Furthermore, most students had a very limited role in information exchange, as manifested by low betweenness and information centrality scores, although being reachable (having moderate closeness centrality).

**Table 2:** Average SNA characteristics of subjects involved in the study. The table lists mean centrality scores in SNA constructs, level of activity and position in information exchange in the three courses before intervention. The reported values of betweenness, closeness, and information were normalized by Gephi.

Construct	Course A	Course B	Course C
**Level of activity**			
Out-degree	17.29	20.12	3.03
In-Degree	0.47	2.73	2.91
Average Degree	17.76	22.85	5.94
**Position in information exchange**			
Betweenness	0.00	0.00	0.01
Closeness	0.99	0.66	0.44
Information	0.06	0.24	0.71

**Table 3:** Roles played by subject in each course. AP = Active participatory, Active Non-participatory = ANP, P = Peripheral.

Course	Role			
	AP	ANP	P	Total
Course A	0	16	1	17
Course B	10	22	1	33
Course C	5	0	27	32
**Total**	**15**	**38**	**29**	**82**

Using the role definitions described earlier in section 2.5 to interpret the different roles played by students; the results tabulated in Table 3 show that only 15 (18.3%) of students were active and participatory, and the majority of roles were not participatory (did not participate in discourse, exchange information or negotiate with others), around third of students were peripheral (rarely participated).

Based on the visualization and quantitative analysis, we can conclude that the three courses had a non-collaborative pattern of interactions, very few interactions among students, and limited information exchange or negotiation. Students’ network of information exchange was very limited and dominated by the teacher.

### The intervention

The intervention aimed at improving the shortcomings in the online collaborative learning regarding the three constructs identified in the monitoring stage (scarce student-student interactions, instructor-centric pattern and poor participation of students in information exchange /negotiation). The intervention was driven by previous recommendations of research in the field of SNA, which called for monitoring and guiding communicational activities as a means to a more efficient collaboration, namely interactivity [19, 20], position in information exchange [8, 22, 26, 27], role in the collaborative group [16, 23, 29], and group cohesion [27, 30]. It was also inspired by evidence that encouraging certain procedures and practices that enhance online collaborative practices would positively enhance learning and improve learning outcomes [2, 3, 11, 12, 45, 46, 56]. The intervention was structured into five actions: increasing awareness, promoting online collaboration, improving the content and content negotiation through scripting, preparing teachers, and finally practicing with feedback. The main part of the intervention was a full day workshop to implement the awareness, training, and practice. The design followed principles suggested by Johnson et al. [57] and Abrami et al [12] through the following steps.

1. **Awareness**: Participants were shown anonymized SNA visualizations and transcripts of the first mid-term online discussions. They commented and reflected upon them and suggested improvements. We stressed the importance of participation of all members in future discussions to their learning and highlighted the social and cognitive benefits of contribution [3].
2. **Collaboration**: Students were prepared and trained for collaboration with a focus on improving group dynamics, boosting interactions among students, and group cohesion [3, 12, 33, 46, 57]. The following concepts were emphasized [57], and later practiced in a sandbox online environment:
  - Attitude: Emphasizing the importance of positive attitude and behavior in the training, namely: 1) Positive interdependence (each participant’s success positively enhances the success of his or her peers); 2) Individual accountability (each participant is responsible for his or her own learning as well as for helping other participants learn.)
  - Promoting interactions: Encouraging participants to help each other analyze and understand the concepts in discussions, share relevant knowledge and resources, promote effective feedback, and work constructively towards a common goal.
  - Group processing and social skills: Training participants to evaluate their own and group contribution to ideas, listening to arguments, and encouraging each other’s participation.
3. **Teacher training**: Teachers were trained on how to facilitate online discourse, engage students in discussions, and stimulate debates [21, 58]. The teachers were encouraged to use clinical cases that are integral to the curriculum objectives, match them to students’ individual learning needs, and motivate the use of prior knowledge, clinical reasoning skills, and argumentation [12].
4. **Content**: Content was improved by using a flexible collaborative script approach; clarifying the objectives, types of activities, sequencing, and explaining roles in order to prevent students from rushing to final conclusions or solving the clinical problem, and to engage the students in a meaningful discourse [14, 20, 39, 40]. Such scripting can rectify the instructor centric role problems by stimulating students to negotiate together and use teachers as facilitators. Scripting might also have a positive effect on students’ position in information exchange [14, 20, 40].
  The online case scenarios were re-structured so that they provided explicit learning objectives; the general objectives were to discuss the clinical reasoning of each case clinical presentation, and how to approach the patient regarding investigations and management plan. Students were advised to start by discussing the clinical findings of the case, the significance of patients’ symptoms and signs, then suggest investigations and proceed later to diagnosis and management. They were advised to debate each other’s approach, provide alternative explanations or plans. Each case scenario was followed by initiating questions about the case, such as “How can you link the case presentation to a certain laboratory finding”, “Should we monitor the patient during treatment and why”, or “what alternative diagnosis you should consider and how to rule it out”. The instructions were flexible regarding specific student roles, the aim was to simulate real life case discussions where in most occasions doctors have comparable roles. Emphasis was laid on participating, following the sequence suggested, offering different perspectives, proposing alternate approaches, and debate/agree with each other’s point of view. Moreover, students were assured that activities are not graded and that reaching a diagnosis is not appreciated better than any other type of interaction. We expect some roles to emerge during collaborative interactions, as students might take the coordinator role and connect different points of views, few others might take the leadership role and the majority to take an active participatory role. Regarding the teachers’ role, they were asked to facilitate, support when asked, and interfere when required in cases of conflict.
5. **Practice and feedback**: Students were allowed to practice in a training online environment, for which they were given feedback and guidance. In addition, a simulation video was published on the front page of the LMS explaining how to participate effectively in a collaborative discussion.

### The second mid-term (post-intervention stage)

#### Visual analysis

**Fig 4:**
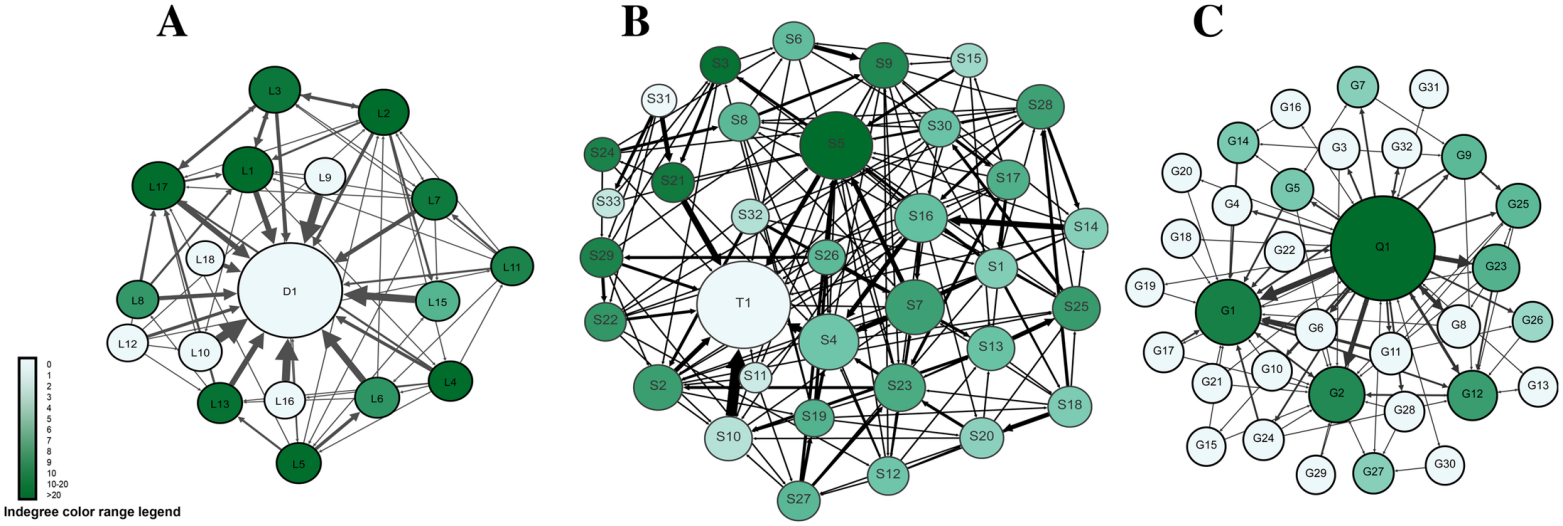
Visualization of all interactions in the three courses post intervention. Each circle (node) corresponds to a participant. Node size is proportional to the degree centrality (sum of incoming and outgoing interactions). Arrows between nodes represent interactions, and the thickness of the arrows represents frequency of interactions. The arrow heads represent the target of interaction. The color range corresponds to betweenness centrality.

The analysis of the second mid-term by means of social network analysis revealed a picture different from the first mid-term, with improvement across the three constructs of SNA that we used as basis of the monitoring strategy, especially level of interactions among students, role in information sharing, and role in groups. As shown in Fig 4, which gives an overview of all interactions in the three courses, there was a marked increase in student-student interactions, reflected in dense and thick interconnections between students’ nodes. Teacher-student interactions also increased significantly; this was most marked in courses C and B, and to a lesser extent in course A. An important development is the appearance of “coordinator students,” who had high betweenness centrality seen as dark green nodes, and who were involved in relaying and connecting other peers.

**Fig 5:**
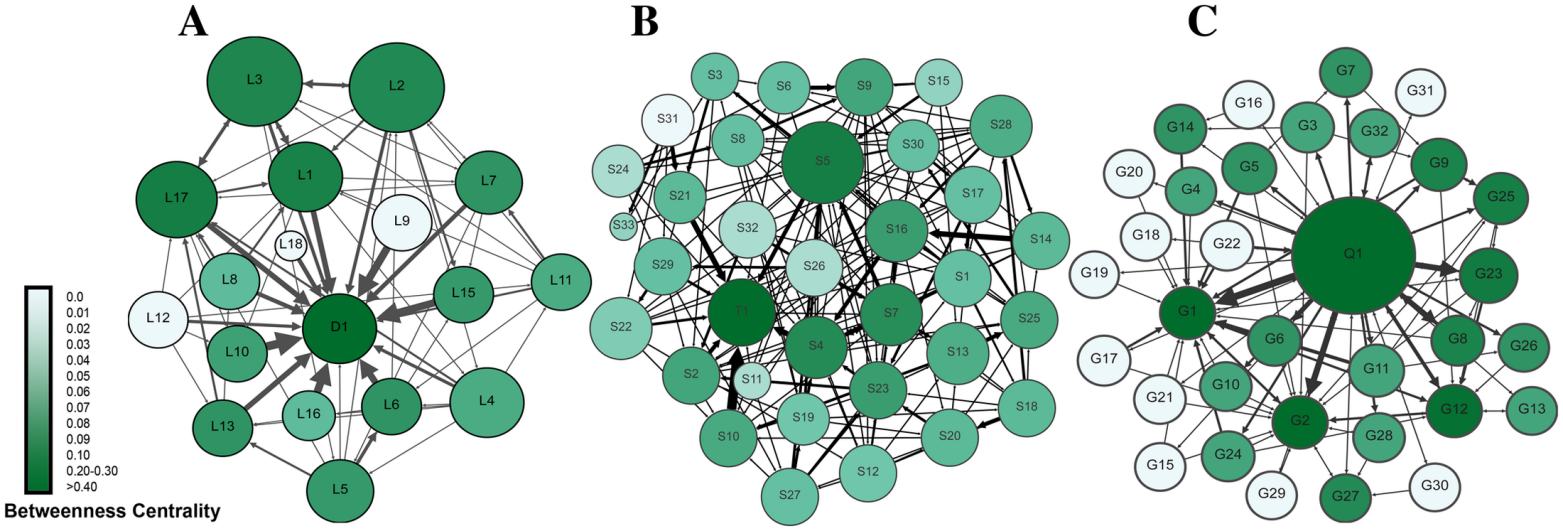
summary of all outgoing interactions in the three courses (information giving network). Each circle (node) corresponds to a participant. Node size is proportional to the out-degree centrality (sum of outgoing interactions). Arrows between nodes represent interactions, and the thickness of the arrows represents frequency of interactions. The arrow heads represent the target of interaction. The color range corresponds to in-degree centrality.

**Fig 6:**
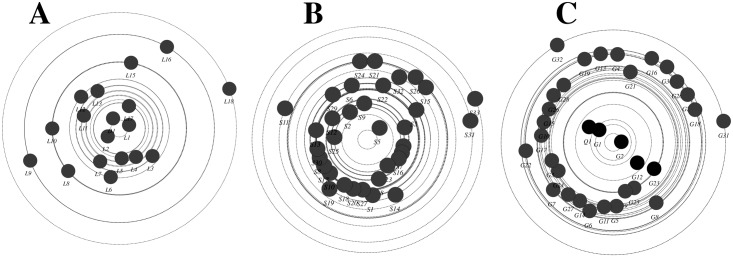
summary of information centrality in all three courses post-intervention. The closer the node is to the center, the more important role it has in information transfer, more students are close to the center compared to the pre-intervention.

Similarly, the information giving network (Fig 5) has improved in a number of ways; compared to the instructor, students’ participation has increased (seen as bigger students’ node sizes indicating higher out-degree centrality). Several students have received more interactions (seen as dark green colored nodes indicating higher in-degree centrality) compared to very few before, indicating more student-student interactions and a distribution of interactions among most members of the groups. In contrast to the first mid-term, Figs 4 and 5 show participatory networks where students interact together, more students play an active role the discussions and mediating information. Regarding information negotiation, the information centrality plot in Fig 6 shows more students closer to the center of the plot in each course, which indicates that more students played a role in information transfer, significantly improving from the first mid-term.

#### Quantitative analysis

Following the visual analyses, a statistical analysis of IA parameters and SNA indicators were performed on both individual and group levels. A comparison was made between the two measurement periods using data from all courses combined as well as on the individual course level.

**Fig 7:**
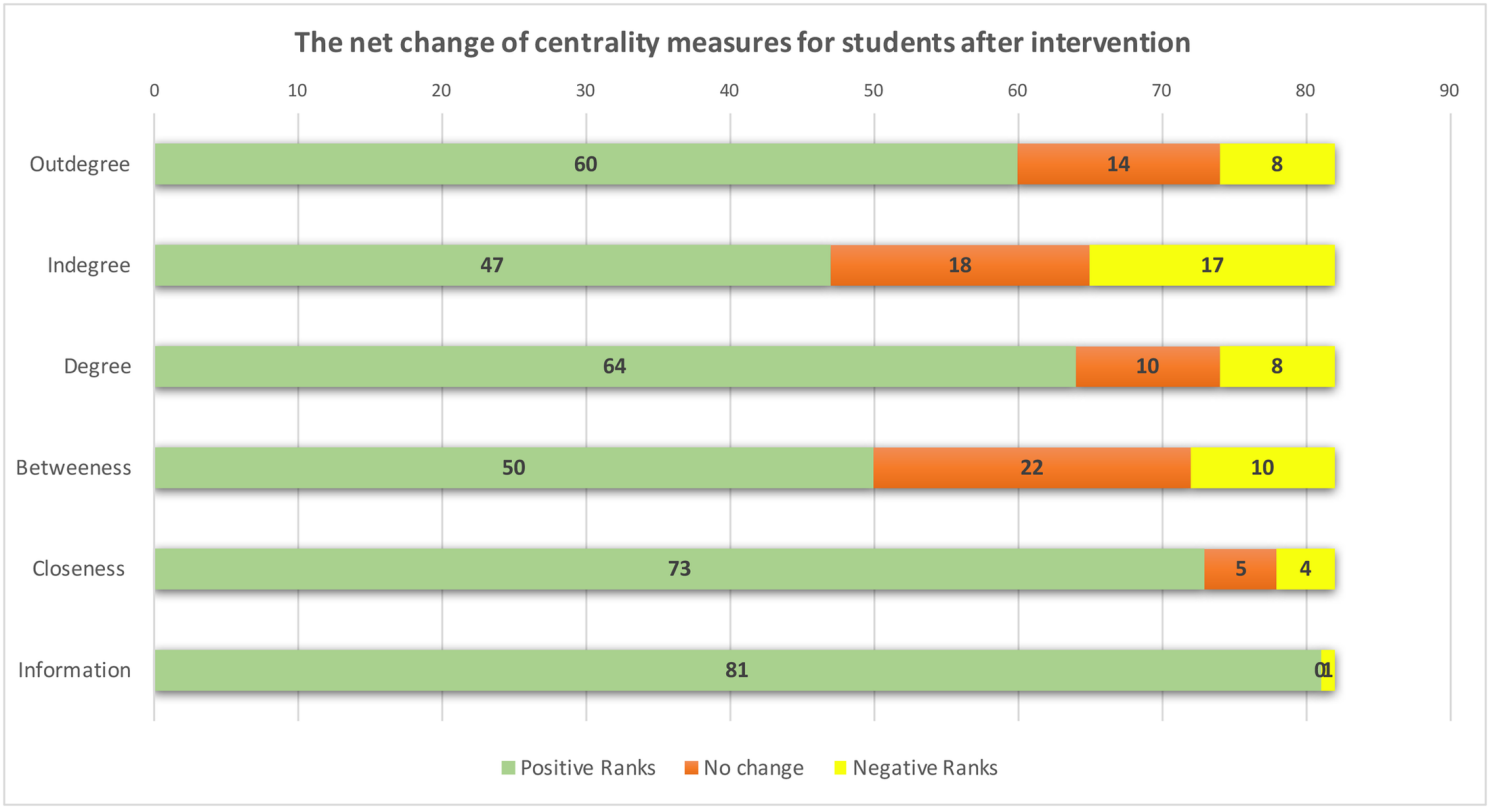
The net change of centrality measures for students after intervention. The figure shows the number of students who had their positions improved, students who did not change and students who declined in the three centrality constructs (Level of activity and position in information exchange). The test compares students’ ranks across the two points of measures (Pre-and post-intervention). A positive rank is an improvement in centrality score, a negative rank is a decline.

Fig 7 compares the centrality measures corresponding to three constructs of SNA, level of activity, role in information exchange. The Shapiro-Wilk test of normality showed that most centrality measures did not follow a normal distribution, so a non-parametric test, Wilcoxon Signed Ranks test, was conducted to compare pre-intervention to post-intervention results. The test ranks students according to their activity level and compares their ranks across two points of measures. The Wilcoxon test was performed on the general level, including data from all students in the three courses combined in Fig 7, as well as on individual course basis. Results of all students showed a statistically significant positive improvement in all measures of centrality across the three constructs. The improvement was more marked in the information exchange indicator; information centrality increased in 98.8% of the students, followed by reachability (closeness centrality). The significant change in information centrality as well as the other centrality measures is an indication of a shift towards efficient information exchange in the network. Full details of all significant centrality measures are shown in Fig 7.

Regarding individual courses, there was a consistent and significant improvement in most of the SNA centrality measures corresponding to role in information exchange. Full details are available in Table 4.

**Table 4:** Centrality measures changes in individual course. The table shows the number of students in each course who had their positions improved, students who did not change and students who declined in the three centrality constructs (Level of activity and position in information exchange). The test compares students’ ranks across two points of measures (Pre-and post-intervention). A positive rank is an improvement in centrality score, a negative rank is a decline.

Course	Centrality	Ranks			p
		Positive (Improved)	Negative (Worsened)	No change	
**Course A**	Degree	12*	4	1	0.008
	In-degree	14*	0	3	0.001
	Out-degree	6	10	1	0.775
	Betweenness	12*	0	5	<0.01
	Closeness	15*	2	3	<0.01
	Information	17*	0	0	<0.01
**Course B**	Degree	1	32*	0	<0.01
	In-degree	22*	4	7	<0.01
	Out-degree	0	33*	0	<0.01
	Betweenness	31*	1	1	<0.01
	Closeness	27*	4	2	<0.01
	Information	33*	0	0	<0.01
**Course C**	Degree	19*	5	8	<0.01
	In-degree	13	12	7	0.397
	Out-degree	21*	5	6	<0.01
	Betweenness	7	9	16	0.134
	Closeness	31*	1	0	<0.01
	Information	31*	1	0	<0.01

* *p*<0.01.

The third construct of SNA we investigated was the roles. The analysis revealed that, in contrast to the first midterm, in each course, more students played an active collaborative role. Moreover, new roles emerged: six leaders and four coordinators compared to none before. There was also a significant increase in active participatory roles from 15 to 40, and consequently decrease in non-participatory roles from 67 to 32 students. Table 5 presents full details on role changes, including per-course roles.

**Table 5:** Roles played by subject in each course after the intervention. L = leader, C = Coordinator, AP = Active, participatory, Active Non-participatory = ANP, P = Peripheral.

Course	Role					
	L	C	AP	ANP	P	Total
Course A	2	1	8	6	0	17
Course B	2	3	20	7	1	33
Course C	2	0	12	6	12	32
**Total**	**6**	**4**	**40**	**19**	**13**	**82**

#### On the group level

There was an improvement in all group properties that reflect the quality of collaboration. The average in-degree centrality, which reflects student-student interactions, increased by 1827.7% in course A, 244.3% in course B and 166.3% in course C. Similarly, the network density, which is a measure of group cohesion, increased by 293.2% in course A, 142.7% in course B and 132.1% in course C. Clustering coefficient, a measure of how much members tend to interact together, also increased significantly in all courses, most markedly in course A by 580%, full details are presented in Table 6. The reason for the change is that students directed all their efforts to their course instructors rather than engage in a constructive discussion. Their replies were often a reiteration of the same contribution of their peers, thinking that teachers evaluate them by how much they participate. This approach was discouraged and students were advised to start by analysis of the clinical case, summarize the important points, elaborate and build on each other’s perspectives, discuss, debate or offer a different approach, relate information to what they learnt and interdependently reach a solution to the problem.

**Table 6:** Comparison of network properties and interaction indicators before and after intervention. The table compares the average values of interaction parameters (Degree, In-Degree, Out-degree) and group cohesion indicators of each course across the two points of measurement. There is a significant increase in cohesion indicators in each course. AV = Average, Co = Coefficient.

Course	Av Degree		Av In-Degree		Av Out-degree		Density		Av Clustering Co	
	Before	After	Before	After	Before	After	Before	After	Before	After
Course A	17.76	**26.24**	0.47	**8.59**	17.29	**17.65**	0.078	**0.23**	0.05	**0.29**
Course B	22.85	14.36	2.73	**6.67**	20.12	7.70	0.08	**0.17**	0.13	**0.21**
Course C	5.94	**8.78**	2.91	**4.84**	3.03	**3.94**	0.14	**0.19**	0.5	**0.67**

The marked increase in in-degree, density and clustering coefficient are signs that interactions in the second midterm included wider range of participants and were more collaborative, groups became more cohesive and cooperative, and many students were a part of the positive change. Degree centrality and out-degree (can also be considered as interaction parameters) were also better in the second mid-term except for course B. However, these are measures of volume of participation and were not considered a target for our intervention.

### Dynamic networks

For a demonstration of the timeline of events that formed the full picture described above in terms of visualization and quantitative analysis, we compiled all interactions in the largest course (course B) along with their respective timestamps in an animated video showing each interaction as it happened in a time-lapse video. In S1 Video, the first midterm (left side) shows a network as it forms, dominated by the teacher with very few student-student interactions. Later, during the course, a similar pattern of interactions continues, but with some exchange between students. The second midterm (left side) shows a participatory network forming from the first day. The teacher participates but does not dominate, interactions are distributed among a wide range of members, the participatory pattern continues throughout the whole course, and the teacher continues to be a moderator, but is no longer the center of all interactions.

To further illustrate the dynamics of interaction at the individual discussion level, the largest discussions of course B were chosen; one from the first midterm and another from the second midterm, since the largest discussions contained many enough interactions to illustrate the patterns. S2 Video shows both discussions side-by-side. The left side shows a clear teacher-centered pattern of interaction. Interestingly the replies are immediate, and all are responses to the teacher. The right side shows a more relaxed rate of interactions, and students take considerable time between each interaction. Many members are participating, S4 and S5 being the most prominent members of the group along with the teacher.

## Discussion

The process of learning analytics is a closed loop that starts by data collection, followed by data analysis to generate insights, which are later used to design interventions or make informed decisions [44, 59, 60]. Closing the loop by creating an appropriate intervention is a key step towards successful learning analytics, and assessing the efficacy of the intervention is another essential step towards improving and refining the whole process [59]. This research study was conducted to assess the value of the full cycle of learning analytics using SNA in monitoring online collaborative learning to diagnose possible gaps or pitfalls, design an appropriate intervention, and test the efficacy of that intervention. The collaboration analysis we used relied mainly on SNA visual and quantitative analysis. Our monitoring included three SNA constructs for each participant, these constructs were first, the level of interactivity of each participant; second the role and position in information exchange and third, the role played by each participant in the collaboration. On the group level, we included interactivity and group cohesion indicators.

Regarding the level of activity, the statistics reported by our interaction analysis showed high level of posts and high activity, seemingly demonstrating sound collaborative activity. However, using SNA proved otherwise. The interactions were not participatory or collaborative. In fact, there were very few interactions among students, limited information exchange or negotiation, and students’ networks were very limited and dominated by the teacher. Using SNA derived insights helped expose the non-participatory interaction patterns, as well as flag some dissatisfactory aspects in collaborative learning that were amenable to intervention. The intervention stimulated student-student interactions, teacher-student interactions, as well as facilitated a collaborative pattern of interactions among students facilitated by the teachers. Ensuring that CSCL are truly interactive and collaborative is a worthwhile effort and deserves the due attention of teachers and learning designers as well. Bernard, Abrami [56] synthesized the evidence from a review of 74 studies and concluded that increasing interactivity between peers, instructors, or content positively affects learning. They reported a significant adjusted average effect of 0.38. They speculated that interaction leads to fostering of internal mental processes, namely meaningfulness and cognitive engagement [56]. Further evidence has been recently reported in a meta-analysis by Borokhovski, Bernard [5], whose primary finding was that courses designed to support collaborative learning by planning activities that promote student-student interaction significantly add to learning [56].

Using SNA to monitor students’ position in information exchange and negotiation has proved useful in uncovering their limited role, which if left unmanaged might affect the collaborative knowledge construction [27, 34, 37]. Our intervention, based on the conducted SNA monitoring, encouraged students to play a more active role, motivated some to moderate discussions and help bond their unconnected peers. Peer moderation of CSCL has affective, cognitive and performance benefits as well as positive effect on engagement and participation [8, 27, 34, 37].

In contrast to IA, which describes roles played by students in terms of quantity. SNA methods helped reveal several dimensions of the collaborative role such as cooperative behavior, brokerage of information, reach and sphere of influence, besides outlining the relation to other collaborators through visualization. Mapping the roles in this study helped identify the ostensibly active however non-participatory roles that were addressed in the intervention. The proper identification and support of roles played by students can greatly enhance the success of the collaboration process, whether the roles were explicitly defined or emerged during interaction [29, 39]. Furthermore, SNA revealed a non-collaborative instructor-centric role; the teacher assumption of a controlling role, where information flows in a teacher-to-student hierarchical mode is expected to affect collaborative learning negatively [11, 58, 61]. A collaborative role can be a moderator, a facilitator, a helper, or a “guide on the side” [7, 11, 21]. Since interaction with teachers can positively influence student academic achievement [56] and predicts good performance [44, 62, 63], it was important to identify and manage the roles played by teachers [12, 21, 58]. The last monitoring target of our study was group interactivity and cohesion. Using SNA indicators of cohesion such as density and clustering coefficient reflects the diversity of contributions and the tendency of group members to work together and externalize their understanding [32]. Since participatory and cohesive groups are stable, efficient and cooperative [22, 32–34], it is an important target for monitoring so that an informed action can be taken when needed [33, 34].

In this study, we used two types of SNA analysis: visual (instant and cumulative at the end of each term) and quantitative analysis. Visualization of interactions offered a convenient general overview of the status of online collaborative learning. The main strengths of visual analysis lied in the extent of information it offered and the ability to quickly render thousands of posts. The analysis was quick to produce, effortless, and updated instantly. It helped to identify a non-collaborative teacher-centered pattern of interactions (hierarchical pattern), map the relations between collaborators and the information exchange networks. The quantitative analysis complemented the visuals by adding a more accurate quantification of level of activity by participants, how they participated in information exchange, their personal networks and offered a platform for identifying the role they played.

These results have implications on different levels. Administrators and instructors need to realize the importance of quality over quantity: high number of discussion posts is not a good sign of collaboration unless those interactions are participatory [6, 9, 11, 22]. SNA enables evaluation of the participatory nature of those discussions so they can meet the intended objectives of the course. For students, automated or semi-automated feedback on their contributions can help improve their performance, enhance their team working skills, foster interdependence, and improve the way they contribute to knowledge construction [3]. For teachers, automatic evaluation of participation quality in a course can be used to inform teachers about problem spots, or to trigger automatic adjustments and interventions, enabling teachers to focus their efforts elsewhere.

Using SNA for evaluating collaborative online learning has its limitations; for example, a reliable automated content analysis could have helped enhance the way we evaluate collaborative learning. However, educational data mining (EDM) tools are difficult to use or interpret by teachers, subject to misinterpretation with no uniform vocabulary or a framework for reporting results [64]. The manual methods require effortful coding and analysis, which may render this time-consuming process challenging to implement, especially for courses with thousands of posts [22]. The methods used to extract SNA data could be considered another limitation; they were not easy to implement and required technical proficiency not available for educators without technical training.

## Conclusions

This study investigated the potential of using social network analysis in monitoring online collaborative learning, finding gaps and pitfalls in application, and the possibility of guiding an informed intervention. SNA-based visualization helped to analyze thousands of discussion posts. The automated SNA visual analysis was quick to produce, updated in instantly, and was easy to interpret. The combination of visual analysis and quantitative analysis enabled us to identify a non-collaborative teacher-centered pattern of interactions in the three courses studied, very few interactions among students, and limited information exchange or negotiation. Students’ network of information exchange was very limited and dominated by the teacher. The information derived from the monitoring enabled us to design a relevant data-driven intervention, and assess its efficacy using experimental, observational, repeated-measurement design. The intervention was able to significantly enhance student-student interactions and teacher-student interactions, improve information exchange, group cohesion as well as achieve a collaborative pattern of interactions among students and teachers. Since efficient, communicative activities are an essential prerequisite for successful content discussion and the realization of the goals of collaboration, we assume that our SNA-based approach can positively affect teaching and learning in many educational domains. Our study offers a proof of concept of what SNA can add to the tools we have to monitor and support teaching and learning in higher medical education.

## Supporting information

S1 Video An animated compilation of interactions in Course B. For a demonstration of the timeline of events that formed the full picture, we compiled all interactions in the largest course, showing each interaction as it happened in a time-lapse video. In S1 video, the first midterm (left side) shows a network as it forms, dominated by the teacher with very few student-student interactions. The second midterm (left side) shows a participatory network forming from the first day.
(MP4)

S2 Video An animated compilation of interactions in two sample discussions. To further illustrate the dynamics of interaction at the individual discussion level, the largest discussions of course B were chosen; one from the first midterm and another from the second midterm, S2 video shows both discussions side-by-side. The left side shows a clear teacher-centered pattern of interaction. The right side shows a more relaxed rate of interactions, and students take considerable time between each interaction. Many members are participating, S4 and S5 being the most prominent members of the group along with the teacher.
(MP4)
